# Effect of Anoxic Atmosphere on the Physicochemical and Pelletization Properties of *Pinus massoniana* Sawdust during Storage

**DOI:** 10.3390/ijerph20010791

**Published:** 2022-12-31

**Authors:** Hongli Chen, Liqiang Zhang, Zhongliang Huang, Zijian Wu, Mengjiao Tan, Xuan Zhang, Longbo Jiang, Xiaoli Qin, Jing Huang, Hui Li

**Affiliations:** 1 College of Mechanical and Electrical Engineering, Central South University of Forestry and Technology, Changsha 410004, China; 2 State Key Laboratory of Utilization of Woody Oil Resource, Hunan Academy of Forestry, Changsha 410004, China; 3 College of Environmental Science and Engineering, Hunan University, Changsha 410082, China

**Keywords:** biomass, anoxic storage, energy consumption, microbial communities, combustion characteristic

## Abstract

The 34-day anoxic storage of *Pinus massoniana* sawdust (PS) in a sealed constant temperature and humidity chambers was carried out to simulate the limited-oxygen storage process inside piles at industrial scale. The effects of anoxic storage on feedstock’s properties and pelletization process were investigated with respect to elemental composition, dry matter loss, thermogravimetric characteristics, energy consumption, pellets’ density, and microbial communities, etc. After anoxic storage, the microbial community of PS samples was altered, such as the fungi content (*Clonostachys*, *Strelitziana*, and *Orbilia*, etc.), resulting the elemental composition of PS was altered. Thus, the cellulose and ash content of the stored PS were increased, while the hemicellulose, volatile, and fixed carbon were decreased. The energy consumption was increased 7.85–21.98% with the increase in anoxic storage temperature and with the additive of fresh soil collected from PS field in storage process. The single pellet density was altered slightly. Meanwhile, the moisture uptake of PS pellets was decreased. After anoxic storage, the combustion behavior of the stored PS became more stable. The results can be applied directly to guide the development of commercial PS storage and pelletization process currently under development in Asia, Europe and North America.

## 1. Introduction

In recent years, reducing CO_2_ emissions has become urgent, due to serious climate change and environmental pollution. Biomass has the advantage of abundance, easy availability, low price and low levels of pollution [1]. It can replace fossil fuels in the blind spots and ends of heating, reducing greenhouse gas emissions. It has thus attracted more and more attention. Data from the European Union shows that biomass resources will account for 27% of total energy consumption by 2030 [2]. The global renewable sources demand will reach 32 × 10^18^ J by 2050 [3]. At the same time, the Chinese government has announced that it will achieve peak carbon usage by 2030 and carbon neutrality by 2060 [4]. Therefore, biomass can play a significant role in reaching global carbon reduction targets. *Pinus massoniana* is a fast-growing forestry stock, which has been widely planted in Central and Southern China. It is rich in lignocellulose, which can be converted into pellets, fuel ethanol, high value chemicals, and various materials after different treatments [5]. As such, *Pinus massoniana* is a satisfying feedstock.

The biomass feedstock has the disadvantages of low bulk density and energy density, which can increase transportation and storage costs [6]. Pelletization is an efficient solution to these problems, as it can produce pellets of uniform shape and size for transportation, storage, and utilization with the higher energy density, higher bulk density, and lower moisture adsorption [7]. Compared with loose biomass feedstock for power generation, the combustion of pellets can improve its combustion efficiency, and be directly used in residential heating stoves and boilers [8].

In general, the market demand for pellets fluctuates seasonally, and demand is higher in winter. The demand for biomass feedstock supply increases in winter, and the purchasing cost is higher than that in summer [9]. Meanwhile, the supply of biomass feedstock is also seasonal, given the specific harvest season for different agroforestry biomass. In order to ensure the logistical stability of biomass feedstock, manufacturers have to collect biomass feedstock during the harvest, and store biomass feedstock for several months, resulting in the necessary storage of biomass feedstock between transportation and utilization. The woody biomass feedstock residues are often stored in large piles indoors or outdoors [10]. Several studies have been performed on the storage of biomass, focusing on the characteristic variations of samples under aerobic storage, dry matter loss and gas emissions during storage period [11]. Tan et al. [12] found that the physical and chemical properties of the *Camellia oleifera* shell changes after aerobic storage, and consequently improves its pelletization behavior and pellet quality. The energy consumption of the pelletized *Camellia oleifera* shell is decreased, while the pyrolysis ability and combustion stability of *Camellia oleifera* shell are improved after aerobic storage. According to Lopes and Steidle Neto, there was a rapid increase in dry matter loss of sugarcane weight during storage, and the rate is higher at higher temperatures [13]. Geronimo et al. found that gas emissions including CO_2_, CH_4_, and N_2_O can be formed from woody residues in a large pile [10]. Therefore, a better understanding of the various changes from the storage process would greatly inform the impacts of using biomass residue for pelletization concerning changes in physical and chemical properties.

Biomass storage piles, which aim to maintain availability of biomass feedstock, are often left undisturbed for months at a time [14]. Krigstin and Wetzel et al. found that the key mechanisms responsible for major changes to woody biomass on storage are (i) living cell respiration, (ii) biological degradation, and (iii) thermo-chemical oxidative reaction [15]. Therefore, the living cell respiration consumes oxygen at the beginning stage of storage. At the same time, oxygen depletion is eventually improved, when biological decomposition occurs in a poorly-ventilated space. These reactions take place and lead to the creation of anoxic conditions. Microbial reactions have occurred already after harvesting within the biomass, mainly caused by fungi species. Some fungi living in biomass can degrade woody biomass polymers and hemicellulose [16,17]. Therefore, anoxic conditions occur in the bottom center of the storage pile leading to changes in microbial communities, which alter the physical and chemical properties of biomass.

Temperature is an indicator of microbial activity [18], and biomass decomposition is most rapid at the appropriate temperature. Suitable temperature and humidity create favorable conditions for further microbial growth in biomass [19].

Biomass is deposited on the land below, when biomass is cut down. When biomass is collected, the soil that gets stuck to the ground when processing waste is collected. Meanwhile, the cutting of fresh biomass, the plant cells were damage and their soluble contents were released [20]. The increase in soluble contents and the addition of soil can cause changes in the composition of the microbial communities and thus affect the dry matter loss and physic-chemical properties of biomass feedstock. Therefore, various reactions change the properties of biomass during anoxic storage. To the best of the authors’ knowledge, few studies have been conducted on the variation of biomass characteristics and its pelletization after anoxic storage. The study aims to measure the properties and pelletization of anoxic stored woody biomass. The following questions are posed:

(1) From the point of economics and practices in *Pinus massoniana* utilization, does the anoxic storage endow any negative effects on the physical and chemical properties of PS?
(2) What are the effects of different microbial community structure on the properties and pelletization of PS after anoxic storage?
(3) Is there any correlation between the anoxic storage and the pyrolysis characteristics?

It is well known that the physical structure and chemical composition will influence the pelletization, pellet properties, and combustion characteristics. To the best of our knowledge, there is a lack of adequate information concerning the changes in physical and chemical properties of *Pinus massoniana* after anoxic storage. In this research, pelletization and pellet properties of stored PS was performed using proximate analysis, ultimate analysis, SEM, dry matter loss and moisture adsorption to evaluate the physicochemical characteristics of PS after anoxic storage. The energy consumption and single pellet density of PS were used to evaluate the pelletization and pellets properties. The kinetic parameters including activation energy was calculated using Flynn-Wall-Ozawa (FWO). The Coats-Redfern (CR) method was also investigated. The results from this research will be helpful in developing PS as fuel products in China.

## 2. Material and Method

### 2.1. Sample Materials

*Pinus massoniana* branches and soil were taken from the demonstration forestland of the Hunan Academy of Forestry in Hunan Province, China. After harvesting, the fresh *Pinus massoniana* branches was cut down and screened to get 10–15 mm particles, and the soil was also grounded to 0.25–0.50 mm. After disposal, the *Pinus massoniana* sawdust (PS) and soil were stored in zip-lock plastic bags at 4 °C for further study.

### 2.2. Experimental Design

The anoxic atmosphere at the bottom of PS storage piles in different seasons was simulated in an industrial-scale storage pile environment. Storage temperature and humidity were selected according to the local climate conditions in four seasons. The storage simulation was conducted in sealed constant temperature and humidity chambers corresponding to three different storage conditions (temperature: 4 °C, 25 °C, and 35 °C, relative humidity: 80%) for 34 days. *Pinus massoniana* branches may get stuck to the ground during harvesting. Therefore, this study established three soil treatment groups with the same temperature and humidity conditions in a sealed constant temperature and humidity chambers. The untreated sample and stored samples were marked as PS, PS-4, PS-25, PS-35, PS-S-4, PS-S-25, and PS-S-35 respectively (Table 1).

After 34 days, PS samples were removed from the sealed constant temperature and humidity chambers. The stored PS samples were treated with deionized water to remove soil from the mixed storage, and then oven-dried at 105 °C for 48 h for later study and determination of dry matter loss. The anoxic stored PS samples were further dried and ground into a 0.125–0.25 mm particle size.

### 2.3. Pelletizing Process

Before pelletization, a certain amount of deionized water was added to the PS particles to obtain a moisture content of approximately 12%, which is the optimal operating condition for pelletization [21]. PS particles were subsequently stored at 4 °C for 48 h to ensure uniformity.

Pelletization was carried out in a DWD-10 pressure unit. The pelleting conditions were as follows: cylinder die temperature was 110 °C, pressure was 4000 N. The pelleting procedure was as follows: the cylindrical die was raised to the preset temperature (110 °C), and after reaching the stable state (30 min), the stored PS particles of about 0.8 ± 0.02 g were filled into the die, which was compressed to 4000 N by the piston at a speed of 5 mm/min, and stayed at 4000 N for 30 s. Subsequently, the plate below the die was removed, and the pellet was extruded from the cylinder with a piston at a rate of 2 mm/min. At the same time, the compaction force, extrusion force, and displacement curves of the feedstock mass were recorded by the computer control system during the pelleting process, and the energy consumption of the feedstock mass was further calculated.

At least ten pellets were produced for each sample. The length and weight of pellets were then measured to calculate single pellet density, and the pellets were stored in a sealed bag for two weeks at 4 °C to examine the released single pellet density.

### 2.4. Physicochemical Properties Analysis

The analysis was performed using an elemental analyzer (Thermo Scientific Flash 2000HT, Milan, Italy) to determine the carbon, hydrogen, and nitrogen contents of the PS. Oxygen content was calculated by difference. The cellulose, hemicellulose, and lignin contents of the PS were determined using a titration method. Proximate analysis was performed using a muffle furnace (SX2-4-10NP, Yiheng, Shanghai, China) and thermogravimetric analyzer (Netzsch-449F3, Waldkraiburg, Germany), which determined the volatile, ash content, and fixed carbon (by difference). The higher heating value (HHV) of PS was analyzed by using an oxygen bomb calorimeter (Sundy Sdacm3100, Hunan Sundy Science and Technology Co.,Ltd., Hunan, China). The morphology of the PS samples was observed using a scanning electron microscope (SEM) (Mira3 lmh, TESCAN, Shanghai, China). The CIELab method for colorimetric analysis was performed using a CR-10 color reader (Konica Minolta Inc, Chiyoda-ku, Japan.).

### 2.5. Moisture Absorption

A constant temperature humidity chamber (HWS-350A) was used to measure the moisture adsorption of PS pellets at 30 °C and 90% relative humidity. Pellets were placed within the chamber and their mass was measured every 20 min for the first five hours. After 48 h, the moisture content of PS pellets was examined as equilibrium moisture content.

### 2.6. Thermogravimetric Analysis

Thermogravimetric and combustion analysis of PS was tested on a thermogravimetric thermal analyzer (Netzsch-449F3, Waldkraiburg, Germany) under nitrogen and air conditions. The average weight of the samples was 10 ± 0.2 mg. Samples of PS was heated from ambient to 800 °C, with heating rates β as 5, 10, 20, and 40 °C/min. The temperature change and weight loss of PS samples during heating process were recorded in thermogravimetric (TG) and derivative thermogravimetric (DTG) formats. The pyrolysis kinetic parameters were studied by CR model and FWO model. The comprehensive combustibility index *S*_N_ was used to characterize the combustion characteristics of samples. The detailed calculation method is introduced through the previous research [22,23].

### 2.7. Microbiological Diversity

The prokaryotic fungal ITS was amplified by PCR with universal primers F: CTTGGTCATTTAGAGGAAGTAA and R: GCTGCGTTCTTCATCGATGC, respectively. The PCR system was 30 μL. The PCR procedure was as follows: 95 °C for 3 min, followed by 27 cycles (30 s at 95 °C, 30 s at 55 °C, and 30 s at 72 °C), and a final extension at 72 °C for 10 min. Six technical replicates were included for the amplification of each DNA sample. The PCR products were purified with a 20% agarose electrophoresis, and all the PCR products were mixed based on equal molarity. Finally, all the purified PCR products were sent to Personalbio Co., Ltd. (Shanghai, China), and double-ended sequencing was performed on the Illumina Miseq platform. The DNA samples from the same ecosystem were sequenced in the same batch. Through clustering, a statistical analysis of biological information was performed on the DADA2 at a 100% similarity level.

## 3. Result and Discussion

### 3.1. Characteristics of the Stored PS

In Table 2, the volatile matter, fixed carbon, and ash content of the untreated PS were 75.01%, 21.62%, and 3.37%, respectively. The ultimate analysis of the untreated PS showed carbon, hydrogen, oxygen, and nitrogen contents of 51.71%, 5.44%, 41.75%, and 1.10%, respectively. After storage for 34 days, the hemicellulose content decreased and the cellulose contents increased. Furthermore, the lignin content of stored PS changed.

Table 2 showed the HHV of untreated PS as 27.04 MJ/kg, and the HHV decreased after anoxic storage. The HHV of PS after anoxic storage mainly due to ash content, the ratio of O/C and H/C, and fixed carbon content [24]. The ash content of untreated PS was 3.37%, while the ash contents of PS-4, PS-25, PS-35, PS-S-4, PS-S-25, and PS-S-35 were 4.38%, 4.73%, 5.07%, 5.26%, 5.92%, and 5.81%, respectively. The ash content, the ratio of O/C and H/C was increased. The decrease in fixed carbon might be the reason for the decreease in HHV during anoxic storage. The HHV of PS is similar to the calorific, which raises the prospect of their potential use as biomass fuel.

**Figure 1:**
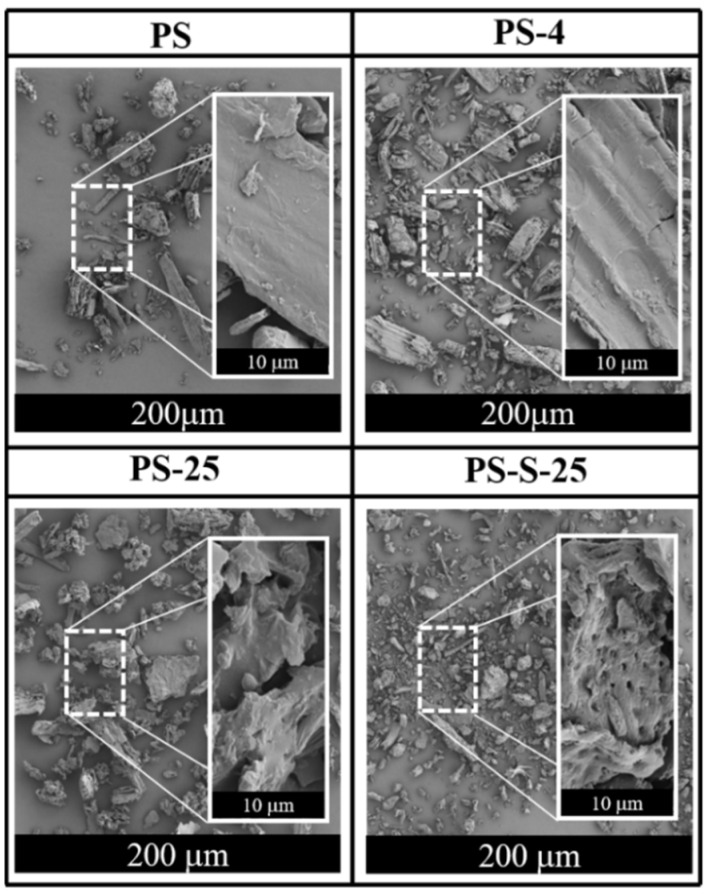
SEM images of PS, PS-4, PS-25, and PS-S-25.

The bulk density of the untreated PS was 218.55 kg/m^3^. Compared with the PS after anoxic storage, the bulk densities of PS-4, PS-25, PS-35, PS-S-4, PS-S-25, and PS-S-35 were increased to 237.06, 240.05, 247.72, 259.31, 269.97, and 260.05 kg/m^3^, respectively (Table 2). Higher bulk density is generally desirable in practice. This is because the denser the material, the lower its storage and transportation cost. In Figure 1, the untreated PS particles were relatively loose and the biomass fiber was relatively smooth. After anoxic storage, the distribution of PS-4, PS-25 and PS-25 is denser and the pore structure is more obvious. The bulk density of the anoxic storage PS samples was increased (Table 2), indicating that the structure of PS particles became more compact after anoxic storage. In sum, the structural of the pellets are consistent with the change of bulk density to some extent. Therefore, the bulk density of PS was increased after anoxic storage, which facilitates subsequent storage and transportation.

**Figure 2:**
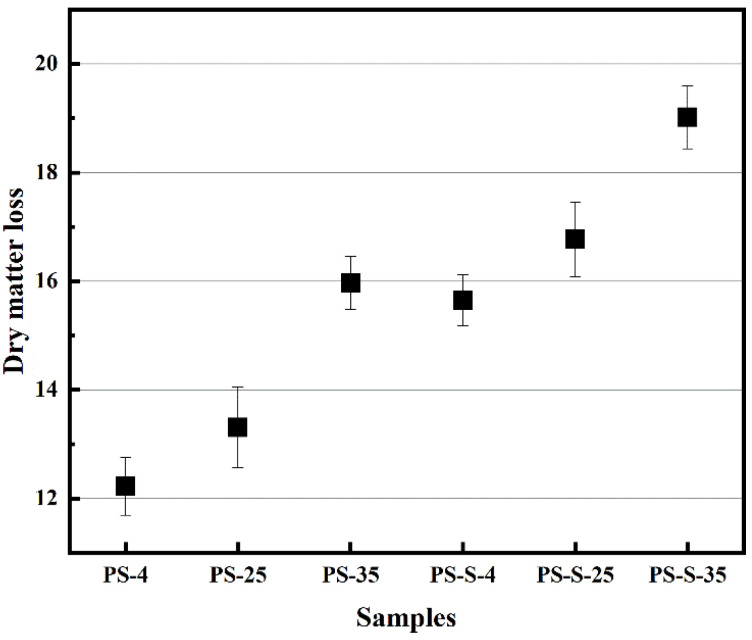
Dry matter loss of the PS after storage under anoxic atmosphere.

As shown in Figure 2, the dry matter loss of PS-4, PS-25, PS-35, PS-S-4, PS-S-25, and PS-S-35 were 12.22%, 13.31%, 15.97%, 15.65%, 16.77%, and 19.02%, respectively. Increasing storage temperature led to the higher dry matter losses. PS-S-35 was decomposed more than PS, PS-4, PS-25, PS-35, PS-S-4, and PS-S-25. The additive of soil enhanced the dry matter loss of samples. All biomass breaks down over time through the same microbial processes. Anoxic degradation leads to increased gas emissions (CH_4_ and N_2_O), resulting in increased dry matter loss. Moreover, the storage temperature may accelerate the decomposition rate. Meanwhile, the dry matter loss was increased at the faster decomposition rate [15]. Initially, the temperature was increased in the pile as the respiration, which promotes the rate of microbial degradation. Leading to the dry matter loss was increase. With the addition of soil, the microbial community became richer, resulting in the increment of dry matter loss of stored PS. The dry matter loss under anoxic storage was decreased, which the biomass feedstock was left greater than that of unstored biomass feedstock. Therefore, the cost of biomass feedstock under anoxic storage was lower than that of unstored feedstock and aerobic storage.

**Figure 3:**
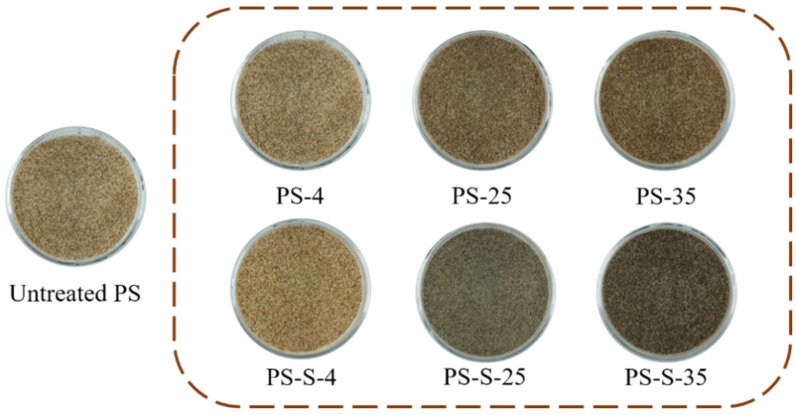
Color change of PS, PS-4, PS-25, PS-35, PS-S-4, PS-S-25, and PS-S-35 particles.

In Figure 3, the brown color was intensified as the storage temperature increasing, and with the addition of soil, the color of the stored PS was dark brown or black. The brightness (L*) of the PS after anoxic storage was all lower than that of the untreated PS. The total color variation (ΔE) was higher than 3 with the decrease from 62.91 to 41.22, which was visible to the naked eye, due to the increase in volatile content, the decrease in hemicellulose content and the degradation of biomass [25].

### 3.2. Pyrolysis and Combustion Characteristics

#### 3.2.1. Thermogravimetric Analysis

**Figure 4:**
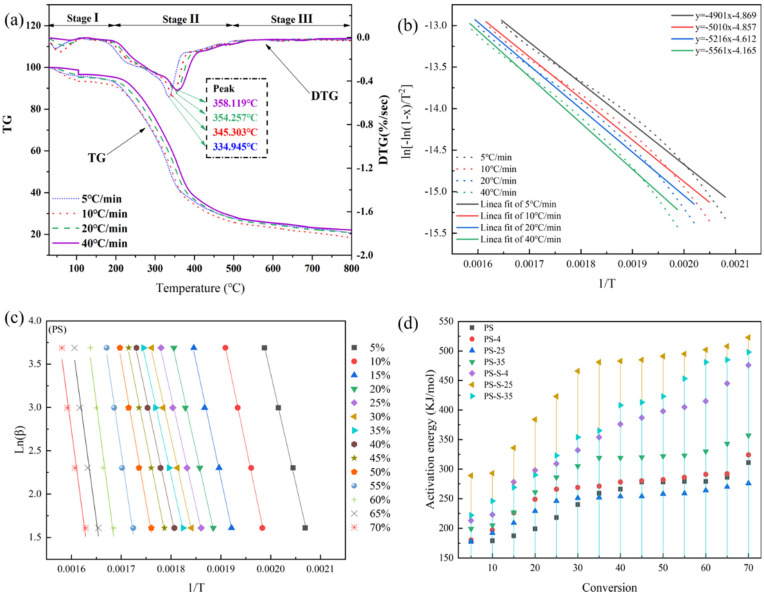
(**a**) TG and DTG curves of PS sample, (**b**) Coats-Redfern model of PS sample, (**c**) Flynn-Wall-Ozawa (FWO) model of PS sample, (**d**) activation energy of PS sample.

Thermogravimetric (TG and DTG) analysis was used to reveal the influence of anoxic storage and soil addition on pyrolysis characteristics of the untreated and anoxic stored PS samples (Figure 4a). According to the TG curve, a three-stage thermal degradation pathway is observed. The first stage corresponds to the loss of free moisture. The mass loss due to water evaporation is about 5%. After that, the curve remains flat until 220 °C. The first stage of the anoxic stored PS is the same as that of the untreated PS.

The second stage (220–600 °C) mainly degrades cellulose, hemicellulose, and lignin et al. A mass loss of nearly 67% was observed at this stage. This phase of untreated PS starts at 220 °C at a heating rate of 5 °C/min. With the further increase in the heating rate (10, 20, and 40 °C/min), the starting temperature of the second stage is increased to 245, 262 and 274 °C, respectively. The same pattern was observed with stored PS (Figure S1, TG and DTG curves of PS-4 (**a**), PS-25 (**c**), PS-35 (**e**), PS-S-4 (**b**), PS-S-25 (**d**), and PS-S-35 (**f**).). The main components of PS are cellulose, hemicellulose, and lignin, for which the thermal decomposition temperature ranges are different. Therefore, the second stage can be further divided into two regions. Firstly, cellulose and hemicellulose are degraded in the temperature range of 270–390 °C. Hemicellulose degrades first at lower temperatures (200–320 °C) due to its low degree of polymerization, and contains many branched chains. Cellulose requires higher energy to break the bonds formed by the long-chain polymers and therefore disintegrates at temperatures over 300 °C. The peak values of DTG curves were 358, 354, 345, and 334 °C, respectively. This is mainly due to the limitation of heat transfer and the temperature gradient between the surface and the interior, affecting the release of volatile matter [26]. Therefore, the two peaks on the DTG curve correspond to the degradation of hemicellulose and cellulose, respectively. The second region is 390 °C–600 °C, representing the thermal degradation of lignin. Lignin contains different aromatic rings with many branches, which results in a wide range of chemical bonds. The third stage is above 600 °C, which is mainly the decomposition of some inorganic substances and ash with the slight mass loss.

#### 3.2.2. Kinetic Model

##### Flynn-Wall-Ozawa Model

The pyrolysis kinetic properties of PS were evaluated by calculating the activation energy (*E*a) by the iso-conversional method Flynn-Wall-Ozawa (FWO). Figure 4c shows the lines for PS determined by the FWO model. Fourteen mass conversion extents from 5% to 70% with 5% increments at each sample heating rate were selected to examine the thermal degradation process. In this study, only the region of greatest weight loss was considered to be related to the pyrolysis fraction of all biomass.

The pyrolysis of PS was described as single-stage kinetic, due to the activation energy having little variation in FWO. The similarity between these values is a strong indication of the validity and conformity of the models used in this study to calculate and unravel the kinetics of thermal decomposition of the residual biomass of PS samples. High values were found for the correlation factor (R^2^) of 0.9935 for the FWO model.

Figure 4c shows the FWO models of PS, PS-4, PS-25, PS-35, PS-S-4, PS-S-25, and PS-S-35, and Figure 4d shows the apparent activation energy (*E*a) of pine at different conversion rates (α) under the FWO model. In PS, *E*a started to be low at the conversion rate of 0.05, which was 199 kJ/mol, and rose to 260 kJ/mol at the conversion rate of 0.50, which increased slowly during this period. When α was greater than 0.50, *E*a increased greatly, and reached 335 kJ/mol with α as 0.70. It can be concluded that the *E*a of PS increased with the increase in conversion α, which may be related to the thermal stability of hemicellulose and cellulose. Lower conversion corresponds to the pyrolysis of hemicellulose at lower temperatures, and higher conversion corresponds to the pyrolysis of cellulose at higher temperatures.

Due to the high content of hemicellulose and cellulose, the minimum activation energy value of PS is about 178 kJ/mol. However, the activation energy values of PS-4, PS-25, PS35, PS-S-4, PS-S-25, and PS-S-35 were 180, 178, 199, 213, 289, and 222 kJ/mol, respectively. The higher value of the activation energy meant greater difficulty for reaction. It can be seen that the apparent activation energy of PS after anoxic storage gradually increased, which is not suitable for commercial application.

##### Coats-Redfern Method

The thermogravimetric data from *T*_a_ to *T*_peak_ were analyzed using the Coats-Redfern method (Figure 4b). The Coats-Redfern model usually provides more information regarding the composition variation during pyrolysis than the FWO model. The activation energy determined using the Coats-Redfern model was much lower than that derived from the FWO model. The activation energies determined by the FWO model were 199–335 kJ/mol, whereas those determined by Coats-Redfern were 51–69 kJ/mol. This is because the FWO model includes the activation energies for both the decomposition reaction and diffusion during pyrolysis, while the Coats-Redfern model only considers the decomposition reaction during pyrolysis. However, both models showed the same overall pattern.

#### 3.2.3. Combustion Characteristics

**Figure 5:**
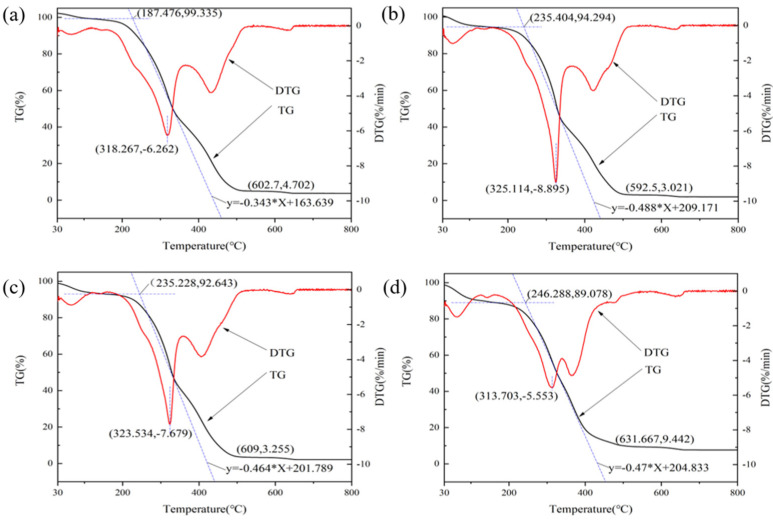
TG and DTG curves of (**a**) PS, (**b**) PS-4, (**c**) PS-25, and (**d**) PS-S-25 at β = 10 °C/min in air atmosphere.

The combustion characteristics of PS at a heating rate of 10 °C/min were analyzed by thermogravimetric analysis under air atmosphere. In Figure 5, the combustion process of the untreated and anoxic stored PS is divided into three stages. The first stage is mainly the volatile matter release. The second stage corresponds to the combustion of carbon, and the third stage was a burnout stage.

According to the TG/DTG curve, some combustion indexes of PS were shown in Table 3, such as combustion characteristic factor (*S*_N_), ignition temperature (*T*_i_), burnout temperature (*T_e_*), flammability index (*C*), and combustion stability (*R*). *T*_i_ of the untreated PS, PS-4, PS-25, and PS-S-25 were 187.48, 256.58, 318.37, and 353.84 °C, respectively, whereas *T*_e_ of the stored PS, PS-4, PS-25, PS-S-25 were 602.70, 607.41, 707.22, and 692.80 °C, respectively. With the increase in anoxic storage temperature and soil addition, the *T*_i_ and *T*_e_ values of PS were increased, indicating that the storage conditions delayed the ignition temperature, likely due to the higher ash content promoting oxygen diffusion and heat transfer. The higher burnout temperature indicated difficulty in burning, requiring longer residence times and higher temperatures to complete combustion. The comprehensive combustibility index (*S*_N_) of PS, PS-4, PS-25, PS-35, PS-S-4, PS-S-25, and PS-S-35 was 67.37 × 10^−8^, 41.31 × 10^−8^, 14.58 × 10^−8^, 16.63 × 10^−8^, 34.78 × 10^−8^, 34.59 × 10^−8^, and 20.46 × 10^−8^, respectively. *S*_N_ is the composite combustion index. The higher the *S*_N_ value, the better the combustion characteristics of feedstock. The *S*_N_ index value was higher than that of coal (8.1 × 10^−8^) [27], indicating that the pellets prepared after anoxic storage have better combustion characteristics. As can be seen from Table 3, C and R of untreated PS were higher than those of PS-4, PS-25 and PS-S-25. The flammability index (*C*) of PS, PS-4, PS-25, PS−35, PS-S-4, PS-S-25, and PS-S-35 was 178.16 × 10^−6^, 111.29 × 10^−6^, 59.94 × 10^−6^, 65.84 × 10^−6^, 71.20 × 10^−6^, and 67.02 × 10^−6^, respectively. The combustion stability (R) of PS, PS-4, PS-25, PS-35, PS-S-4, PS-S-25, and PS-S-35 was 6.01, 5.63, 2.70, 3.57, 3.54, and 3.33, respectively. *C* and *R* were stable combustion indexes, which reflect the stability of combustion. The lower the values of these indexes, the more stable the pellet combustion process. The reason for the decrease in *C* and *R* was owing to hemicellulose degradation during anoxic storage.

### 3.3. Pellet Properties

#### 3.3.1. Energy Consumption

**Figure 6:**
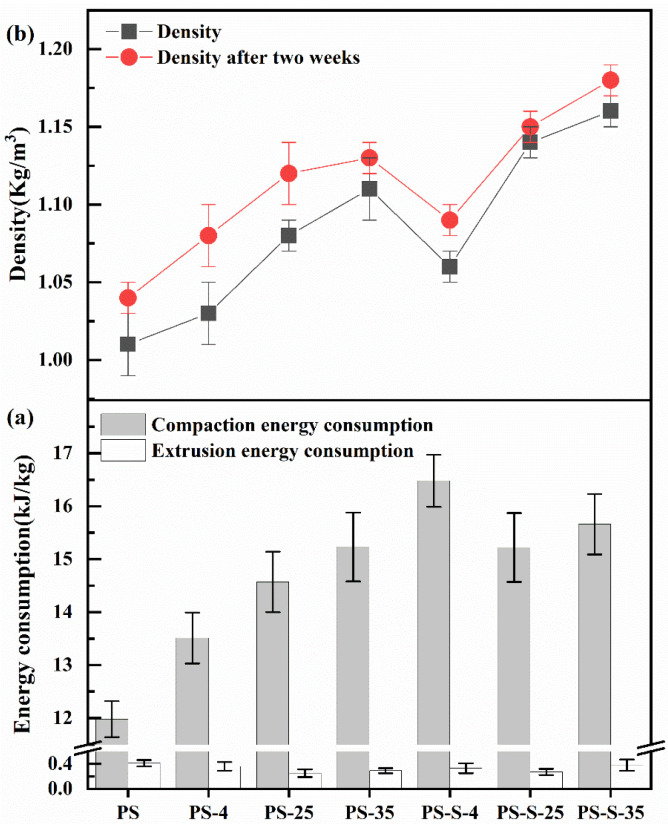
(**a**) Energy consumption for pelletization, (**b**) single pellet density of PS, PS-4, PS-25, PS-35, PS-S-4, PS-S-25, and PS-S-35.

As shown in Figure 6a, the compaction energy consumption of PS, PS-4, PS-25, PS-35, PS-S-4, PS-S-25 and PS-S-35 were 13.51, 14.57, 15.23, 16.48, 15.22, and 15.22 kJ/kg, respectively. As result, the compaction energy consumption of PS after anoxic storage was higher than that made from the control PS group. The compaction energy consumption comes from the static friction between pellet and die. Hemicellulose, lignin, protein, starch, sugar and fat are natural adhesives in the pelleting process and are triggered at temperatures ranging from 75 to 150 °C [28]. After anoxic storage, the decreased relative content of hemicellulose in PS reduced the plasticity of PS, which consequently increased the compaction energy consumption. In addition, the decreased glass transition temperature (*T*_g_) of lignin was improved by water molecules spreading on the surface and inner section of the particles during storage [12], which increased particle plasticity and reduced compaction energy consumption. In addition, microorganisms consume the lipids in the community. Lipids could reduce friction in the pelleting process of materials and promote the relative movement between material particles, so the energy consumption of lipid-rich substances is reduced [29]. The microorganisms become more active after anoxic storage, which could consume more lipids. Thus, the compaction energy consumption of stored PS was increased.

After compaction, the relaxation of the pellets from the die was caused by the incomplete plasticity of the pellets, which can be identified by the extrusion energy consumption. For the untreated PS (11.98 kJ/kg), the extrusion energy consumption of the stored PS decreased to 0.36, 0.25g, 0.29, 0.33, 0.27, and 0.38 kJ/kg, respectively. The increment in temperature and soil addition resulted in a significant increase in the pelleting energy consumption of pelletization for the samples after anoxic storage. During pelletization, the friction among the PS particles and die wall caused the lignin to be heated and softened, forming a wax layer on the pellet surface and decreasing the extrusion energy consumption. In this study, the remarkable increment of compaction energy consumption of PS after anoxic storage. Furthermore, the energy consumption of PS group with soil mixed were increased. Therefore, manufacturers should avoid storage in anoxic environment and avoid staining the soil.

#### 3.3.2. Single Pellet Density

Single pellet density is the main index of pellet quality. For the single pellet density of untreated PS (1.01 g/mm^3^), the single pellet density after anoxic storage increased to 1.03, 1.18, 1.11, 1.06, 1.14, and 1.16 g/mm^3^, respectively. It can also be determined from Figure 6 that the single pellet densities of the PS pellet in all runs were decreased after two weeks. Single pellet density is correlated with biomass hemicellulose. The hemicellulose of PS pellets after storage decreases, the lower the elasticity, the larger the pores, and thus the lower the single particle density. In addition, the PS contains a large amount of ash (3.37–5.81%), which may cause weak bonds between the particles, as well as a very low internal bond in the pellets. As result, the density of individual particles was decreased as the hypoxic storage progressed and storage temperature increased, which may reduce the pellet quality decreases after anoxic storage.

#### 3.3.3. Moisture Absorption

**Figure 7:**
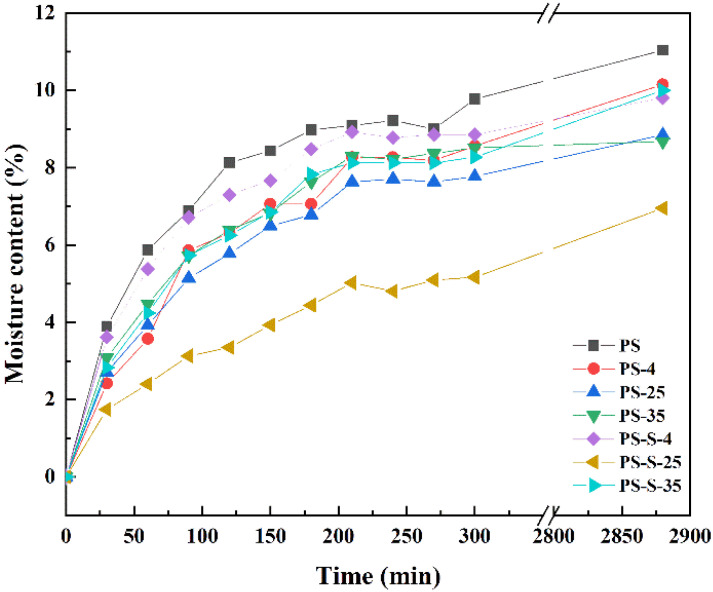
Moisture absorption of PS, PS-4, PS-25, PS-35, PS-S-4, PS-S-25, and PS-S-35 samples.

As shown in Figure 7, the moisture absorption of the untreated PS pellets was higher than the stored PS samples. After 48 h, the moisture content of all PS pellets kept equilibrium. For the untreated PS pellet, the moisture content is 11.04%. The anoxic stored sample of PS-4, PS-25, PS-35, PS-S-4, PS-S-25, and PS-S-35 were 10.15%, 8.85%, 8.68%, 9.81%, 6.95%, and 9.99%, respectively. This result indicated that the moisture absorption of PS samples decreased obviously after anoxic storage, which is convenient for storage and transportation subsequently. This is mainly because hemicellulose is rich in hydroxyl group, which is the main factor affecting the moisture absorption of biomass pellets. Moreover, the hydrogen bonds and solid bridges were loosed in the PS pellets after moisture absorption, which leads to burst into flames during storage and transportation [30]. Therefore, the water absorption of PS pellets after anoxic storage decreases with the decrease in hemicellulose, which increases the security of storage and transportation.

### 3.4. Differences in Fungi Communities via Anoxic Storage

#### 3.4.1. The Diversity of the Fungi Communities

By clustering, the biological information on DADA2 was statistically analyzed at a 100% similarity level. As shown in Table S1 (Alpha diversity index of PS, PS-25, and PS-S-25), the high coverage value close to one showed that the results are credible. The microbial richness represented by the Chao1 index decreased from 299 to about 251 by PS and PS-25, indicating the richness of fungi community decreasing after anoxic storage. The Chao1 index of PS-25 and PS-S-25 increased from 251 to 268, showing that richness of fungi community increased after soil addition [31]. The Shannon index evaluating the diversity degree of microbial community also showed a similar tendency, as PS-25 embraced the highest value of Shannon, while untreated PS showed the lowest Shannon value at around 4.22. In sum, the richness and diversity of the PS fungi community were significantly decreased after anoxic storage and soil addition. Therefore, it can be inferred that the high temperature and soil addition during the anoxic storage unfavored the growth and development of some dominant species, and reduced the structural stability of microbial communities.

**Figure 8:**
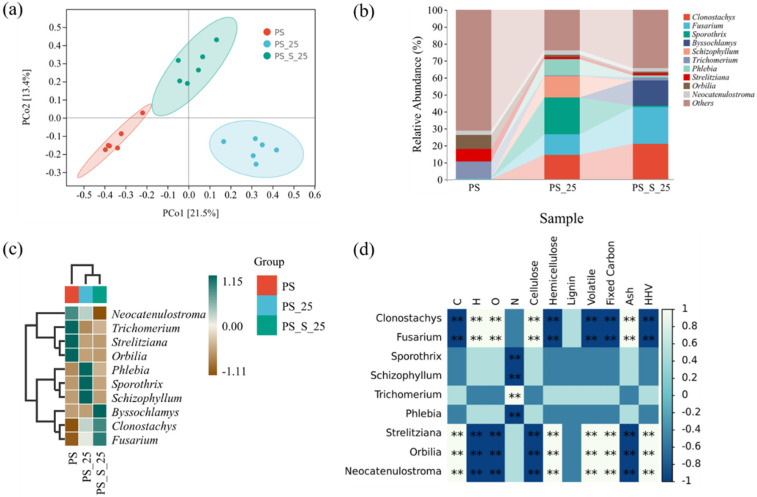
Fungi community analyzes conducted during PS, PS-25, PS-S-25 groups. (**a**) PCoA of the fungi communities in the compost based on DADA2, (**b**) Fungi level composition of the fungi community, (**c**) Heat map of species composition, (**d**) Corheatmap. ** There is a correlation, white is positive, dark blue is negative.

To further reveal the fungi community structure, the principal coordinates analysis (PCoA) was conducted based on the Bray-Cutis distance, except for the application of α diversity. PCo1 and PCo2 accounted for 34.9% of the total variation (Figure 8a). Figure 8a showed that the community structure of the PS samples was divided into three different profiles corresponding to different treatment group, affirming that the anoxic storage and soil addition played a significant role in fungi community of stored PS.

#### 3.4.2. The Structure of the Fungi Communities

Figure 8b shows the relative percentages at the genus level. Species with an abundance less than 0.05 in all samples were classified as other species. The main genera of the untreated group PS were *Trichomerium* (10.06%), *Strelitziana* (7.52%), and *Orbilia* (8.04%). The main genera of PS-25 were *Clonostachys* (14.11%), *Fusarium* (12.37%), *Sporothrix* (21.78%), *Schizophyllum* (12.64%) and *Phlebia* (9.72%). The main genera of PS-S-25 were *Clonostachys* (20.63%), *Fusarium* (22.07%), and *Byssochlamys* (14.54%). It was found that the composition of the dominant species in PS, PS-25, and PS-S-25 groups was not similar, while the dominant species after anoxic storage were similar, such as *Clonostachys* and *Fusarium*.

As shown in Figure 8d, the color ranges from brown to dark green to indicate the degree to which the species plays an important role in the biomass. Darker shades of dark green indicate higher correlations, and darker browns indicate lower correlations. In the untreated PS group, *Orbilia*, *Trichomerium,* and *Strelitziana* played an important role. *Phlebia*, *Sporothrix*, and *Schizophyllum* played an important part at PS-25 group. *Byssochlamys*, *Fusarium*, and *Clonostachy* became the dominant bacteria for PS-S-25.

Fungi play an important role in the degradation of biomass in nature, which can produce hydroxyl radicals to attack the cell wall of biomass or produce cellulase and hemicellulase through oxidation reactions. The cellulase and hemicellulose produced by the enzyme hydrolysis reaction can convert cellulose and hemicellulose in biomass into monomeric sugar [32]. The content of cellulose and hemicellulose will affect the pelletization and pellet properties.

#### 3.4.3. The Relationship between PS Characteristics and the Fungal Community

In Figure 8d, the corheatmap analysis were carried out to assess the relationship between PS characteristics (C, H, O, N, cellulose, hemicellulose, lignin, volatile, fixed carbon, ash, and HHV) and the fungal community, to clarify the extent of the influence of fungal community on PS characteristics and determine which fungi were most influential. Among all fungi, *Strelitziana*, *Orbilia*, and *Neocatenulostroma* had the greatest impacts on hemicellulose, volatile, C, fixed carbon, and HHV. *Clonostachys* and *Fusarium* had the greatest impacts on cellulose, ash, H, and O. The research of Gomes et al. indicated that *Clonostachys* has the potential to produce cellulases and pectinases, which are necessary for the degradation of plant biomass feedstock. *Clonostachys* appeared in PS-25 and PS-S-25 after anoxic storage, which produce cellulases and pectinases to decompose the cellulose [33]. The research of Sun et al. mentioned that the *Strelitziana* was the dominant genera in biomass, which could decompose the hemicellulose [34]. Therefore, it can be inferred that *Strelitziana*, *Orbilia*, *Neocatenulostroma*, *Clonostachys* and *Fusarium* were the main factors affecting the PS characteristics, and the results prove that there was an important relationship between PS characteristics and the fungal community.

## 4. Conclusions

This study investigated the effect of anoxic storage on PS properties and pelletization. The dry matter loss and bulk density of PS increased after anoxic storage. Compared with the untreated PS, with the increment of anoxic storage temperature and whether soil is added for storage, the flammability index of PS was decreased, showing that the combustion safety improved significantly. The compaction energy consumption of PS was increased after anoxic storage. With the increment of anoxic storage and soil addition, the moisture content of PS pellets was decreased. The richness of microbial community on PS was decreased after anoxic storage. The pelletization and pellet properties are better under aerobic environment. The results showed that the anoxic storage is not favourable for physicochemical and pelletization properties. Therefore, the anoxic storage of biomass at high temperature should be avoided in the actual storage process. The results can be applied directly to guide the development of commercial PS storage and pelletization process currently under development in Asia, Europe and North America.

## Figures and Tables

**Table 1:** The conditions of PS samples.

Samples	Conditions
PS	The untreated PS sample
PS-4	PS stored at 4 °C
PS-25	PS stored at 25 °C
PS-35	PS stored at 35 °C
PS-S-4	PS and understory soil were mixed 1:1 and stored at 4 °C
PS-S-25	PS and understory soil were mixed 1:1 and stored at 25 °C
PS-S-35	PS and understory soil were mixed 1:1 and stored at 35 °C

**Table 2:** The proximate and ultimate analysis of untreated PS and the stored PS after 34 days storage.

Analysis	PS	PS-4	PS-25	PS-35	PS-S-4	PS-S-25	PS-S-35
Elemental analysis (wt%)							
C	51.71	47.28	46.49	45.84	43.74	43.51	43.27
H	5.44	5.96	5.93	6.02	6.21	6.56	6.74
O	41.75	45.82	46.79	47.06	49.32	48.92	49.17
N	1.10	0.94	0.79	1.08	0.73	1.01	0.82
O/C	0.81	0.97	1.01	1.03	1.13	1.12	1.14
H/C	0.11	0.13	0.13	0.13	0.14	0.15	0.16
Chemical composition (wt%)							
Cellulose	32.63	34.80	35.11	33.14	34.03	35.39	39.94
Hemicellulose	15.43	12.56	11.28	12.86	13.04	11.12	10.37
Lignin	24.52	24.56	23.23	25.58	22.18	25.63	24.58
Proximate analysis (wt%)							
Volatile	75.01	75.14	74.69	73.94	74.51	73.81	74.74
Fixed Carbon	21.62	20.48	20.58	20.99	20.23	20.27	19.45
Ash	3.37	4.38	4.73	5.07	5.26	5.92	5.81
HHV(MJ/kg)	27.04	24.43	23.33	23.65	24.32	23.27	23.85
Bulk density (kg/m^3^)	218.55	237.06	240.05	247.72	259.31	269.97	260.05

**Table 3:** The flammability index (*C*), comprehensive combustibility index (*S*_N_) and combustion stability (*R*) of PS samples.

Samples	*T*_i_ (°C)	*T*_e_ (°C)	(dw/dt) _Mean_	*C*	*S*_N_	*R*
PS	187.48	602.70	2.28	178.16 × 10^−6^	67.37 × 10^−8^	6.01
PS-4	256.58	607.41	2.25	111.29 × 10^−6^	41.31 × 10^−8^	5.63
PS-25	318.37	707.22	1.72	59.94 × 10^−6^	14.58 × 10^−8^	2.70
PS-35	290.95	723.36	1.83	65.84 × 10^−6^	16.63 × 10^−8^	2.79
PS-S-4	305.47	604.89	2.65	79.52 × 10^−6^	34.78 × 10^−8^	3.57
PS-S-25	353.84	692.80	2.88	71.20 × 10^−6^	34.59 × 10^−8^	3.54
PS-S-35	345.72	709.67	2.17	67.02 × 10^−6^	20.46 × 10^−8^	3.33

## Data Availability

The data is unavailable due to privacy or ethical restrictions.

## Supplementary Materials

The following supporting information can be downloaded at: https://www.mdpi.com/article/10.3390/ijerph20010791/s1, Table S1: Alpha diversity index of PS, PS-25, and PS-S-25; Figure S1: TG and DTG curves of PS-4 (**a**), PS-25 (**c**), PS-35 (**e**), PS-S-4 (**b**), PS-S-25 (**d**), and PS-S-35 (**f**); Figure S2: FWO model of PS-4, PS-25, PS-35, PS-S-4, PS-S-25, and PS-S-35.
