# Supplementary material for: Effect of Anoxic Atmosphere on the Physicochemical and Pelletization Properties of Pinus massoniana Sawdust during Storage
Source: Int J Environ Res Public Health. 2022 Dec 31;20(1):791. doi: 10.3390/ijerph20010791 (PMC9819789; doi:10.3390/ijerph20010791)
Supplement: Supplementary file 1 [file ijerph-20-00791-s001.zip › ijerph-2082323-supplementary.pdf]

Table S1 Alpha diversity index of PS, PS-25, and PS-S-25.

Sample	Chao1	coverage	Shannon	Simpson
PS-1	301.448	1.00	4.64962	0.857271
PS-2	232.093	1.00	5.58035	0.958484
PS-3	264.45	1.00	4.54754	0.909132
PS-4	352.956	1.00	5.5054	0.938808
PS-5	272.499	1.00	5.2818	0.938011
PS-6	367.717	1.00	6.33909	0.975474
PS-25-1	200.551	1.00	3.84212	0.840311
PS-25-2	271.536	1.00	4.06688	0.836964
PS-25-3	273.166	1.00	4.9836	0.930516
PS-25-4	348.165	1.00	5.81863	0.960603
PS-25-5	120.066	1.00	1.55693	0.371674
PS-25-6	292.061	1.00	5.06545	0.93601
PS-S-25-1	274.622	1.00	4.58696	0.889002
PS-S-25-2	317.625	1.00	4.90735	0.874864
PS-S-25-3	234.589	1.00	4.00043	0.836443
PS-S-25-4	183.949	1.00	1.99579	0.454452
PS-S-25-5	319.719	1.00	5.87671	0.962188
PS-S-25-6	279.658	1.00	4.92238	0.925322

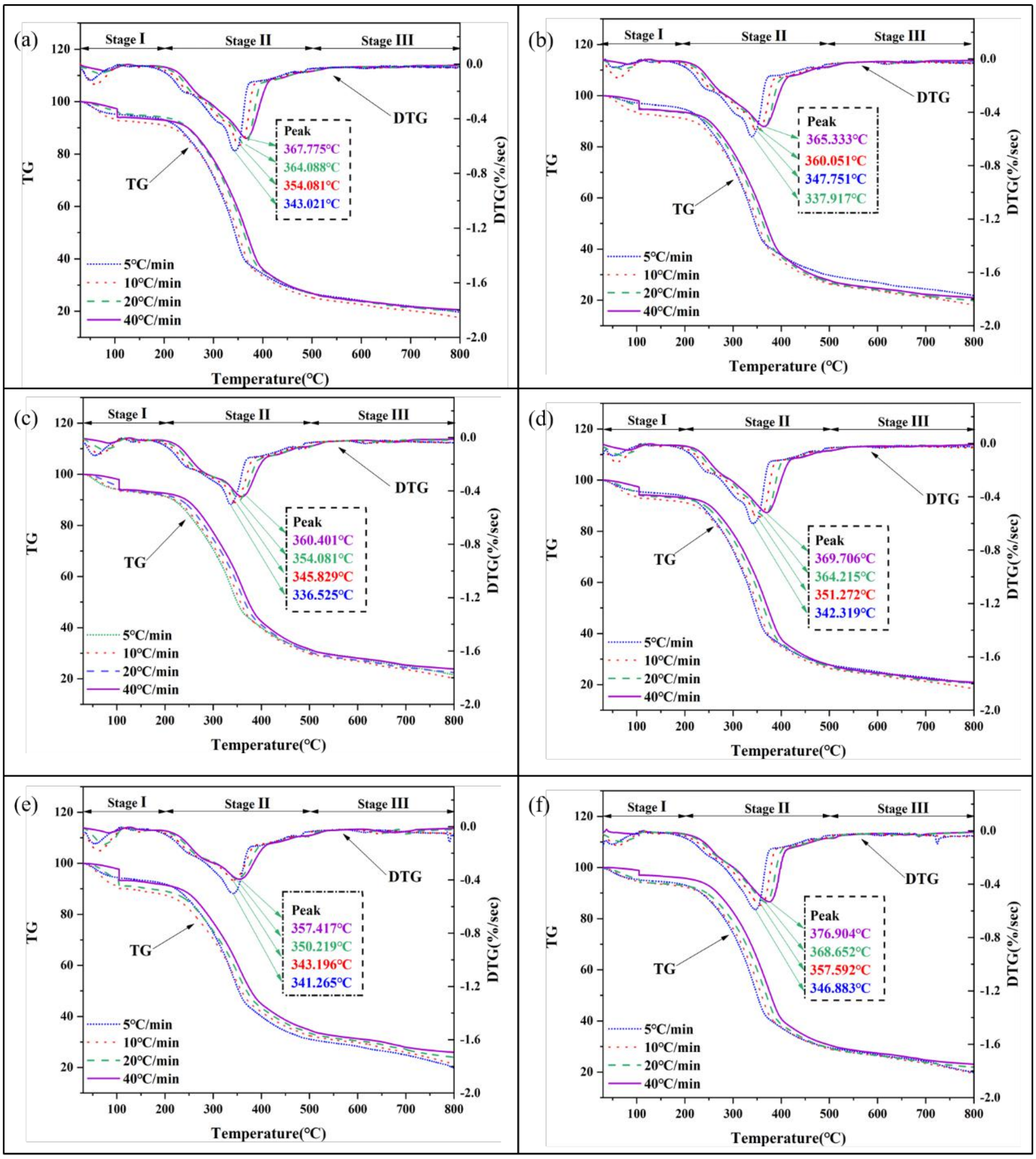

Figure.S1. TG and DTG curves of PS-4, PS-25, PS-35, PS-S-4, PS-S-25, and PS-S-35.

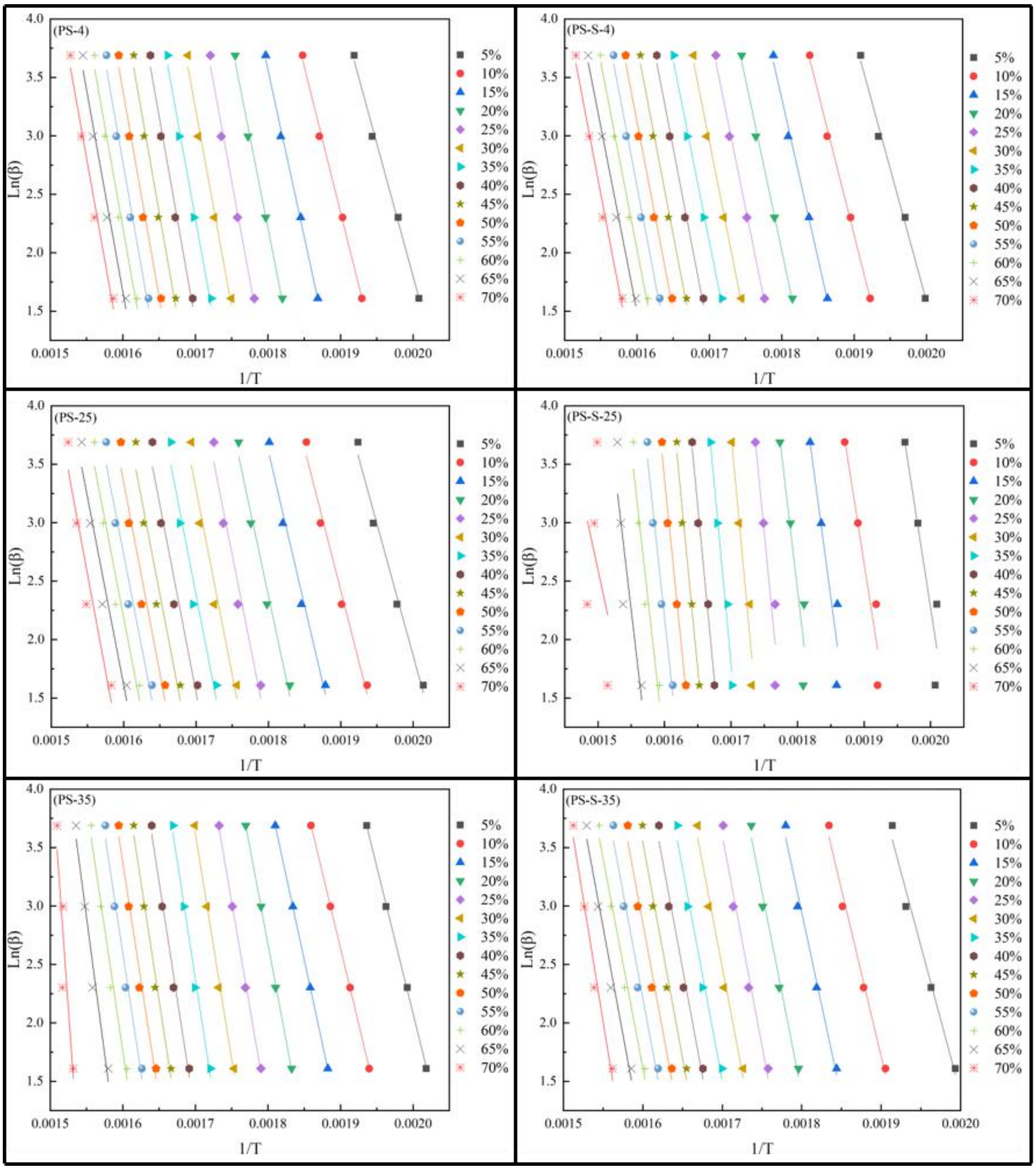

Figure.S2. FWO model of PS-4, PS-25, PS-35, PS-S-4, PS-S-25, and PS-S-35.
